# Neo-adjuvant chemotherapy followed by surgery and chemotherapy or by surgery and chemoradiotherapy for patients with resectable gastric cancer (CRITICS)

**DOI:** 10.1186/1471-2407-11-329

**Published:** 2011-08-02

**Authors:** Johan L Dikken, Johanna W van Sandick, HA Maurits Swellengrebel, Pehr A Lind, Hein Putter, Edwin PM Jansen, Henk Boot, Nicole CT van Grieken, Cornelis JH van de Velde, Marcel Verheij, Annemieke Cats

**Affiliations:** 1 Department of Surgery, K6-R, Leiden University Medical Center, P.O. Box 9600, 2300 RC Leiden, The Netherlands; 2 Department of Radiotherapy, Netherlands Cancer Institute - Antoni van Leeuwenhoek Hospital, Plesmanlaan 121, 1066 CX Amsterdam, The Netherlands; 3 Department of Surgery, Netherlands Cancer Institute - Antoni van Leeuwenhoek Hospital, Plesmanlaan 121, 1066 CX Amsterdam, The Netherlands; 4 Department of Oncology, Stockholm Söder Hospital, Karolinska University Hospital - Huddinge, SE-118 83 Stockholm, Sweden; 5 Department of Medical Statistics, Leiden University Medical Center, P.O. Box 9600, 2300 RC Leiden, The Netherlands; 6 Department of Gastroenterology and Hepatology, Netherlands Cancer Institute - Antoni van Leeuwenhoek Hospital, Plesmanlaan 121, 1066 CX Amsterdam, The Netherlands; 7 Department of Pathology, VU University Medical Center, P.O. Box 7057, 1007 MB Amsterdam, The Netherlands

## Abstract

**Background:**

Radical surgery is the cornerstone in the treatment of resectable gastric cancer. The Intergroup 0116 and MAGIC trials have shown benefit of postoperative chemoradiation and perioperative chemotherapy, respectively. Since these trials cannot be compared directly, both regimens are evaluated prospectively in the CRITICS trial. This study aims to obtain an improved overall survival for patients treated with preoperative chemotherapy and surgery by incorporating radiotherapy concurrently with chemotherapy postoperatively.

**Methods/design:**

In this phase III multicentre study, patients with resectable gastric cancer are treated with three cycles of preoperative ECC (epirubicin, cisplatin and capecitabine), followed by surgery with adequate lymph node dissection, and then either another three cycles of ECC or concurrent chemoradiation (45 Gy, cisplatin and capecitabine). Surgical, pathological, and radiotherapeutic quality control is performed. The primary endpoint is overall survival, secondary endpoints are disease-free survival (DFS), toxicity, health-related quality of life (HRQL), prediction of response, and recurrence risk assessed by genomic and expression profiling. Accrual for the CRITICS trial is from the Netherlands, Sweden, and Denmark, and more countries are invited to participate.

**Conclusion:**

Results of this study will demonstrate whether the combination of preoperative chemotherapy and postoperative chemoradiotherapy will improve the clinical outcome of the current European standard of perioperative chemotherapy, and will therefore play a key role in the future management of patients with resectable gastric cancer.

**Trial registration:**

clinicaltrials.gov NCT00407186

## Background

In the Western world, most patients with gastric cancer present with advanced stages of disease, leading to a low 5-year survival of around 25% [1,2]. After surgical resection, the majority of patients will develop a locoregional recurrence [3]. Many different strategies have been evaluated to improve the outcome of gastric cancer surgery. Randomized trials investigating the role of a more extended lymph node dissection (D2) in comparison with the standard D1 lymphadenectomy, found no difference in overall survival, while a D2 dissection was associated with increased postoperative mortality and morbidity [4-7].

Two Western studies have changed current clinical practice in the treatment of resectable gastric cancer. The Intergroup 0116 study showed a significant benefit in overall survival with adjuvant chemoradiotherapy (CRT) consisting of 45 Gy of radiotherapy combined with fluorouracil (5-FU), and leucovorin, compared to surgery alone [8]. In the British MAGIC (Medical Research Council Adjuvant Gastric Infusional Chemotherapy) study, a significant overall survival benefit was found favouring perioperative chemotherapy (epirubicine, cisplatin, and continuous 5-FU infusion, ECF-regimen) versus surgery alone [9].

Taken the abovementioned pivotal studies together, the important question that needs to be answered is whether postoperative chemoradiotherapy improves survival as compared to postoperative chemotherapy in patients who are treated with neoadjuvant chemotherapy followed by gastric resection. Due to differences in study design and eligibility criteria between the Intergroup 0116 and the MAGIC study, comparing results of these trials is intrinsically not possible (Table 1). Therefore, the two regimens should be compared in a prospective, randomized manner. This is performed in the currently accruing CRITICS trial (ChemoRadiotherapy after Induction chemoTherapy In Cancer of the Stomach). In the present manuscript, we describe the study protocol of this trial and reflect on the possible implications.

**Table 1:** Comparison of Intergroup 0116, MAGIC and CRITICS trials

	**Intergroup 0116**[8]	**MAGIC**[9]	CRITICS
**General**			

Accrual	1991 - 1998	1994 - 2002	2007 -

Number of patients	556	503	788 (needed)

Randomization	after R0 surgery	after diagnosis (before any treatment)	after diagnosis (before any treatment)

**Inclusion**			

Histology	Adenocarcinoma	Adenocarcinoma	Adenocarcinoma

Location	GOJ/Stomach	Lower 1/3 oesophagus/GOJ/Stomach	GOJ (bulk tumour in stomach)/Stomach

Stage	IB-IV (M0)	II-IV (M0)	IB-IV (M0)

**Preoperative Therapy**			

Preoperative therapy	not applicable	A: ECF (3 courses) B: none	A: ECC/EOC (3 courses) B: ECC/EOC (3 courses)

Completed preoperative therapy	not applicable	86%	ongoing

**Surgery**			

Surgery	D0 gastrectomy: 54% D1 gastrectomy: 36% D2 gastrectomy: 10%	oesophagogastrectomy: 23% D1 gastrectomy: 19% D2 gastrectomy: 40% non-curative/unknown: 18%	ongoing

R0 resection	100% (if R1/R2: no inclusion)	A: 69.3% B: 66.4%	ongoing

**Postoperative Therapy**			

Postoperative Therapy	A: 5-FU/LV/RT (45Gy) B: none	A: ECF (3 courses) B: none	A: CC/RT (45Gy) B: ECC/EOC (3 courses)

Completed postoperative therapy	64%	42%	ongoing

**Quality Assurance**			

Surgery	D2 recommended Postoperative analysis of extent of LN dissection	not reported	D1+ resection Regular feedback to individual surgeons and pathologists

Radiotherapy	Central review of RT plan Major deviations corrected	not applicable	Central review of at least first 3 RT plans of each center CTV contouring atlas available

**Results**			

Primary endpoint	Overall Survival	Overall Survival	Overall Survival

Result primary endpoint (experimental versus control)	A: 42% 5-year OS B: 25% 5-year OS	A: 36% 5-year OS B: 23% 5-year OS	ongoing

A: experimental arm

B: control arm

GEJ: gastro-oesophageal junction

5-FU: 5-fluorouracil

LV: leucovorin

RT: radiotherapy (always 25 × 1.8 Gy in 5 weeks)

CC: capecitabine/cisplatin

ECC: epirubicin/cisplatin/capecitabine

EOC: epirubicin/oxaliplatin/capecitabine

CTV: clinical target volume

GOJ: gastro oesophageal junction

OS: overall survival

D1+ resection: removal of stations 1-9 and 11, at least 15 lymph nodes, no routine splenectomy

R0: microscopically radical

## Methods/design

### Study design and objectives

**Figure 1:**
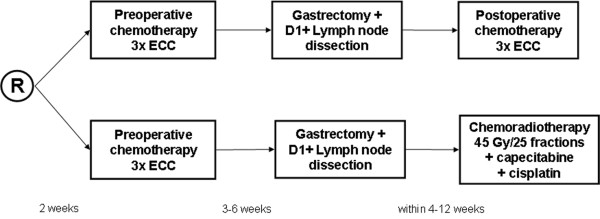
**Randomization scheme**. R: randomization. ECC: epirubicin, cisplatin, capecitabine.

The CRITICS study is an international, multicentre, randomized phase III trial. The primary objective is to compare overall survival between patients treated with neoadjuvant chemotherapy followed by surgery and either postoperative chemotherapy or postoperative chemoradiotherapy for resectable gastric cancer (Figure 1). Secondary endpoints include disease-free survival, toxicity, health-related quality of life (HRQL), prediction of response and recurrence risk assessed by genomic and expression profiling. Randomization is performed directly after entering the study, before the administration of preoperative chemotherapy.

The study started in January 2007 and as of May 2011, 350 patients have been included, while a total of 788 is required to meet the H0 hypothesis that the experimental arm with adjuvant chemoradiotherapy improves OS by 10% or more. In the first two years only a few centres in the Netherlands included patients in this trial. At current times, about 50 centres are collaborating, and, besides the Netherlands, Sweden and Denmark are participating countries (clinicaltrials.gov NCT00407186).

### Patient selection and preoperative staging

Patients with histologically proven stage Ib-IVa (AJCC 6^th ^edition) gastric adenocarcinoma are eligible for this study. The gastro-oesophageal junction (GEJ) may be involved, but the bulk of the tumour has to be in the stomach. Patients should be at least 18 years old and WHO performance status should be 0 or 1. Patients must have adequate haematological, renal and liver functions as defined in the study protocol. Left ventricular ejection fraction should not be lower than 50%.

Exclusion criteria include: previous malignancy, inoperability due to technical surgery-related factors or general condition, and a solitary functioning kidney within the potential radiation field.

Baseline investigations consist of blood tests, an oesophagogastroduodenoscopy with tumour biopsy samples, computed tomography (CT) of the chest and abdomen, renography, cardiac ejection-fraction scan, electrocardiography, and when the preoperative CT-scan suggests peritoneal carcinomatosis, diagnostic laparoscopy. Endoscopic ultrasonography and a PET-scan are optional.

Randomization is performed with stratification for Lauren classification (intestinal, diffuse, or mixed type adenocarcinoma, or unknown), localization (GEJ, proximal, mid, or distal stomach) and hospital.

### Preoperative chemotherapy

Within two weeks after randomization, preoperative chemotherapy is started. All patients are treated with 3 cycles of epirubicin, cisplatin, and capecitabine (ECC). Epirubicin 50 mg/m^2 ^and cisplatin 60 mg/m^2 ^are administered on day 1 intravenously every three weeks, with adequate hydration. Capecitabine is given orally on days 1-14 in a dose of 1000 mg/m^2 ^bid. In Sweden, oxaliplatin 130 mg/m^2 ^is administered instead of cisplatin in order to facilitate chemotherapy administration in the outpatient clinic setting without the need for prehydration. At the start of the study no reimbursement was available for oxaliplatin in the treatment of gastric cancer in the Netherlands.

Response evaluation with CT-scan after two cycles of chemotherapy is aimed primarily to identify patients with early progression.

### Surgery

Surgery is planned 3-6 weeks after the last chemotherapy course. The definitive decision to proceed to surgery is taken based on the absence of signs of progressive disease and an ASA classification of 1 or 2.

Under general anaesthesia supported by epidural anaesthesia, a midline laparotomy is performed, followed by a complete exploration of the abdomen including peritoneal surfaces, liver, and in women, the ovaries. Any free abdominal fluid is aspirated for cytological examination. A curative resection is not possible in case of tumour infiltration into the head of the pancreas requiring a Whipple procedure, para-aortic lymph node metastases below the renal arteries, tumour positive cytology of free peritoneal fluid, or peritoneal metastases that cannot be included in the planned local resection. If curative resection is not possible, the best palliative surgical option is to be decided upon by the surgeon.

**Figure 2:**
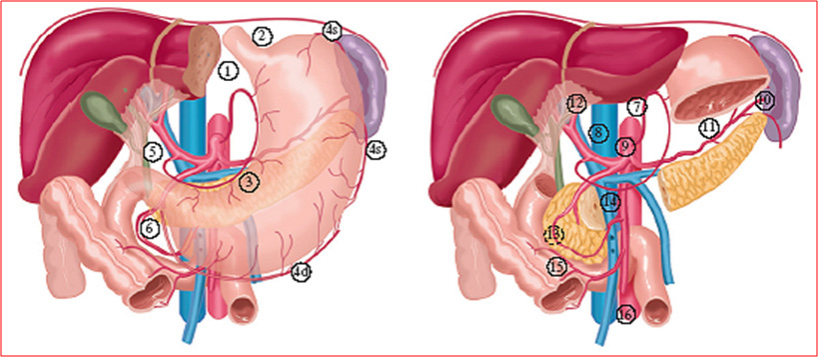
**Lymph node stations as defined by the Japanese Research Society for Gastric Cancer**. right cardial nodes 1. left cardial nodes. 2. along the lesser curvature. 3. along the greater curvature. 4. suprapyloric nodes. 5. infrapyloric nodes. 6. along the left gastric artery. 7. along the common hepatic artery. 8. around the celiac axis. 9. at the splenic hilum. 10. along the splenic artery. 11. in the hepatoduodenal ligament. 12. dorsal to the pancreatic head. 13. at the root of the mesentery. 14. in the traverse mesocolon. 15. para-aortic nodes.

Principle of surgery is a wide resection of the tumour bearing part of the stomach (total, subtotal or distal gastrectomy) en bloc with the N1 and N2 lymph nodes (stations 1-9 and 11, Figure 2) [10] with a minimum of 15 lymph nodes, without routine splenectomy and resection of the pancreatic tail (D1+ lymph node dissection). If possible, a macroscopic proximal and distal margin of 5 cm should be obtained. Adjacent organs are only removed when there is suspicion on tumour involvement.

The continuity of the gastrointestinal tract is restored by a Billroth II reconstruction or with the use of a Roux-en-Y loop. Whether the anastomosis is hand-sutured or stapled is left up to the surgeon. A feeding jejunostomy is strongly advocated and is left *in situ *until postoperative treatment has been completed and oral intake is adequate.

### Pathology

The specimen is sent to the pathologist, preferably fresh and unopened to enable the collection of fresh frozen tissue, followed by processing and reporting of the specimen according to the study protocol. The pathology report includes a minimal dataset containing the following items: type of tumour, localization and size of tumour, invasion depth, surgical margins, and number of (tumour positive) lymph nodes. All specimens undergo additional central pathology review for grading of histological response [11].

### Postoperative treatment

Between 4-12 weeks following surgery, patients in the control arm are given another 3 courses of ECC. Patients in the experimental arm are treated with radiotherapy combined with capecitabine and cisplatin during five weeks. Capecitabine in this group is administered in a dose of 575 mg/m^2 ^bid from Monday to Friday. Cisplatin is administered at a dose of 20 mg/m^2 ^intravenously with pre- and posthydration weekly. The chemotherapy doses are based on previous dose-finding studies in The Netherlands Cancer Institute [12,13] (see discussion).

Radiotherapy consists of 45 Gy in 25 fractions of 1.8 Gy with a frequency of five fractions a week. External beam therapy is used to irradiate the tumour bed, anastomoses and regional lymph nodes. The *clinical target volume *(CTV) has to be delineated on CT-images based on all diagnostic information available.

In defining a *planning target volume *(PTV), the CTV has to be expanded in all directions with a margin of 10 mm, except towards the vertebrae and kidneys, where a margin of 5 mm is applied. All 3D conformal (or IMRT, *intensity modulated radiotherapy*) techniques are allowed to get a homogeneous dose distribution in the PTV. AP-PA techniques are judged to be suboptimal and are therefore not allowed.

Target volume delineation manuals and workshops are offered to all participating radiation oncologists. A digital CTV contouring atlas is made available for all local investigators by the study coordinators. Furthermore, all centres are asked to provide CTV contouring and treatment plans of the first three included patients (or of consecutive patients if considered necessary) to the study coordinators before start of treatment, as interobserver variability in CTV delineation for postoperative radiotherapy after gastric resection is large [14].

### Toxicity and adverse events

Toxicity is measured according to NCI Common Toxicity Criteria (CTC), version 3.0. When preoperative chemotherapy is postponed for more than two weeks consecutively, chemotherapy should be discontinued and the patient should proceed to surgery when possible. Dose modification rules are defined in the study protocol [15].

Serious adverse events are defined according to the rules of good clinical practice and must be reported within one working day.

### Follow-up

After treatment, patients are followed by a medical oncologist or gastroenterologist (and radiation oncologist when they received radiotherapy) on a monthly basis during the first three months, followed by three-monthly visits during the rest of the first year and visits every six months until five years of follow-up. Beyond the initial postoperative period, follow-up by the surgeon is planned every 6 months. CT-scanning and renography is performed every 6 months, followed by yearly scans after 2 years of follow-up.

### Statistics

Based on results from the Intergroup 0116 [8] and MAGIC [9] trials, it is estimated that 5-year overall survival in the perioperative chemotherapy group is 40% and in the chemoradiotherapy group 50%. In order to detect a difference between 40% and 50% in 5-year overall survival with a power of 80% and a significance level of 0.05, about 430 events are required, which corresponds to a total of 788 patients.

Data analysis will be performed according to the intention to treat principle.

An interim analysis is performed when half of the required number of events have been observed.

### Ethics

All patients receive both oral and written information about the study. Randomization can only take place when patients have signed an informed consent. The study is carried out in agreement with the declaration of Helsinki. The study has been approved by the Medical Ethical Committee of the Netherlands Cancer Institute - Antoni van Leeuwenhoek Hospital.

### Quality assurance

Local monitoring has been performed for the first three patients in the first ten participating centres and continuation of the monitoring will be performed.

Furthermore, surgical and pathological quality is monitored for every patient, and feedback to the individual surgeons and pathologists on their own performance is used to improve surgical and pathological quality.

### Side studies

Patients fill out quality of life questionnaires EORTC QLQ-C30 and STO22 five times after randomization: before treatment, after preoperative chemotherapy, after surgery, after postoperative therapy and during follow-up after 12 months.

After finishing accrual and survival analysis, the value of the Maruyama Index of unresected disease [16] and the Memorial Sloan-Kettering Cancer Center (MSKCC) predictive nomogram [17] will be investigated. Furthermore, collected tumour tissue and serum will be used for genomic profiling and further translational research focussing on prognostic and predictive biomarkers.

## Discussion

### Surgery

In both the British MRC trial [4,5] and the Dutch Gastric Cancer Trial (DGCT) [6,7] that randomized gastric cancer patients for a D1 or D2 lymph node dissection, overall survival was not statistically different between the two groups, while a D2 dissection was associated with increased postoperative mortality and morbidity. This might be partially attributed to the higher number of splenectomies and pancreatectomies with a D2 dissection. Another study showed that splenectomy is associated with a twofold risk of postoperative complications [18].

Therefore, it is suggested that performing a gastrectomy with dissection of at least 15 (N1 and N2) lymph nodes, but without routine splenectomy and resection of the pancreatic tail, a so called D1+ resection, can result in a better outcome [19]. The rationale for a minimum of 15 nodes has been the observation that patients with at least 15 nodes examined have superior survival as compared to patients with fewer nodes examined [20,21].

While the Intergroup 0116 study, which had no strict surgical quality protocol, was criticized for its low number of per protocol prescribed D2 dissections [16], in the MAGIC study the percentage of D2 dissections was higher, although no surgical or pathological quality measurements were performed. In the CRITICS study, the Maruyama Index (MI) of unresected disease is used to estimate surgical quality [16]. Also, feedback to individual surgeons and pathologists on their own performance is used to improve surgical and pathological quality.

### Postoperative chemoradiotherapy

The Intergroup 0116 study is the key trial supporting the use of postoperative chemoradiotherapy in the potentially curative treatment of gastric cancer [8]. Because of this trial, postoperative CRT is currently a standard option in the United States for patients undergoing curative resection of stage Ib-IV gastric cancer [22]. However, the study has been criticized because it had no strict surgery and pathology quality protocol, suboptimal surgery (with 54% D0 resections while at least a D1 resection should be recommended), a complex, toxic and nowadays outdated chemotherapy schedule with minimal room for interaction with the daily radiation sessions, and the fact that patients were highly selected (only R0 resections with adequate postoperative recovery). In addition, toxicity in the chemoradiotherapy arm was substantial, with only 64% of the patients completing the planned treatment. In a Dutch retrospective study, postoperative chemoradiation after a D2 dissection was not associated with improved survival [23], in contrast to the results of a large observational Korean study [24].

Since the Intergroup 0116 study was initiated in the early 90s, the concept of concurrent chemoradiotherapy has nowadays been further developed. Capecitabine, an oral prodrug of 5-FU, mimics continuous infusion of 5-FU, and has proven its feasibility in combination with cisplatin and radiotherapy in several phase I/II studies in advanced, resectable gastric cancer [12,25], while its systemic exposure was not found to be compromised by the radiation treatment [26]. In these studies, acute toxicity was low, and compliance to the treatment protocol was high (89-100%). The maximum tolerable doses that evolved from these studies are currently used in the CRITICS study. Renal toxicity was addressed in a prospective fashion, showing a reduction in contribution of the left kidney to total renal function in more than half of the patients, especially after 2D radiotherapy techniques [27]. This illustrates the need for precise modern radiotherapy techniques to minimize renal toxicity.

### Chemotherapy

Many studies have been performed with adjuvant chemotherapy in resectable gastric cancer. These studies have been part of several meta-analyses, which could demonstrate no, or at the most a modest survival benefit for adjuvant chemotherapy [28-33]. Newer chemotherapy schedules, with capecitabine and oxaliplatin, have shown to be as least as effective as schedules with 5-FU and cisplatin, with respect to overall survival (REAL-2 study) [34].

The combination of adjuvant with neo-adjuvant chemotherapy has proven its value in two randomized studies. In the MAGIC study, perioperative chemotherapy resulted in a reduction of the tumour stage, a 10% higher resectability rate and a significant survival benefit of 13% at 5 years [9]. It should be noted that only 55% started postoperative chemotherapy and 42% of the patients completed the entire treatment. The major reasons for a premature treatment stop were tumour progression, postoperative complications, patients' refusal and toxicity. A French prospective trial showed comparable results with 48% of the patients completing the total regimen [35]. The final report of this study has to be awaited. A recent EORTC study comparing preoperative chemotherapy and D2 surgery with D2 surgery alone was stopped early because of poor accrual. A higher R0 resection rate was found in the chemotherapy arm, but no benefit in survival was detected in this underpowered study [36].

Due to the strong position of perioperative chemotherapy with tumour downsizing and downstaging the CRITICS investigators were reluctant towards a randomization arm without preoperative chemotherapy. Therefore, both arms have the same preoperative chemotherapy schedule. This also leads to comparable resection rates thus eliminating the effect of surgery (and preoperative therapy) on a potential survival difference between the two treatment arms.

### Future perspectives

With the CRITICS trial, several other studies on the treatment of resectable gastric cancer are ongoing or have just finished. In the currently accruing MAGIC-B study, patients are randomized between perioperative ECC courses with or without bevacizumab. In the Korean ARTIST trial, which finalized accrual, patients were randomized between postoperative chemotherapy with cisplatin and capecitabine versus chemoradiotherapy after a D2 gastric resection. No preoperative therapy was administered. Feasibility data of this study were reported at ASCO-GI 2009 showing good toxicity profiles with compliance rates of 75% versus 82% respectively. Survival data of this trial have to be awaited [37].

An interesting development is the use of trastuzumab for Her2 positive tumours, which has shown an impressive survival benefit in metastasized gastric cancer [38]. This raises the question if trastuzumab is a valuable addition to the currently used chemotherapy regimens for Her2 positive, resectable gastric cancer. But so far, no such trials have been initiated.

### Final remarks

Accrual for the CRITICS study has been expanded to Sweden and Denmark and more countries are invited to participate. It is expected that the results of this study will play a key role in the future treatment of patients with resectable gastric cancer [39].

## Competing interests

The authors declare that they have no competing interests.

The study is partly funded by an unrestricted grant from Roche. Roche Netherlands has no role in study design, data collection, analysis, interpretation, the writing of the manuscript, or the decision to submit the current study.

## Authors' contributions

JD drafted the manuscript, JvS, EPMJ, HB, NvG, CJHvdV, MV and AC co-authored the writing of the manuscript. All authors participated in the design of the study and approved the final manuscript.

## Pre-publication history

The pre-publication history for this paper can be accessed here:

http://www.biomedcentral.com/1471-2407/11/329/prepub
